# Differential effects of Usutu and West Nile viruses on neuroinflammation, immune cell recruitment and blood–brain barrier integrity

**DOI:** 10.1080/22221751.2022.2156815

**Published:** 2023-01-02

**Authors:** Orianne Constant, Ghizlane Maarifi, Jonathan Barthelemy, Marie-France Martin, Bachirou Tinto, Giovanni Savini, Philippe Van de Perre, Sébastien Nisole, Yannick Simonin, Sara Salinas

**Affiliations:** a Pathogenesis and Control of Chronic and Emerging Infections, INSERM, University of Montpellier, Etablissement Français du Sang, Montpellier, France; b CNRS, Institut de Recherche en Infectiologie de Montpellier, Université de Montpellier, Montpellier, France; c Istituto Zooprofilattico Sperimentale dell'Abruzzo e del Molise (IZS-Teramo), Teramo, Italy; d INSERM, Pathogenesis and Control of Chronic and Emerging Infections, University of Montpellier, Etablissement Français du Sang, CHU Montpellier, Montpellier, France

**Keywords:** Usutu virus, West-Nile virus, blood-brain barrier, innate immunity, neuro-inflammation, leukocytes, immune cell binding

## Abstract

Usutu (USUV) and West Nile (WNV) viruses are two closely related *Flavivirus* belonging to Japanese encephalitis virus serogroup. Evidence of increased circulation of these two arboviruses now exist in Europe. Neurological disorders are reported in humans mainly for WNV, despite the fact that the interaction and effects of viral infections on the neurovasculature are poorly described, notably for USUV. Using a human *in vitro* blood–brain barrier (BBB) and a mouse model, this study characterizes and compares the cerebral endothelial cell permissiveness, innate immunity and inflammatory responses and immune cell recruitment during infection by USUV and WNV. Both viruses are able to infect and cross the human BBB but with different consequences. We observed that WNV infects BBB cells resulting in significant endothelium impairment, potent neuroinflammation and immune cell recruitment, in agreement with previous studies. USUV, despite being able to infect BBB cells with higher replication rate than WNV, does not strongly affect endothelium integrity. Importantly, USUV also induces neuroinflammation, immune cell recruitment such as T lymphocytes, monocytes and dendritic cells (DCs) and was able to infect dendritic cells (DCs) more efficiently compared to WNV, with greater propensity for BBB recruitment. DCs may have differential roles for neuroinvasion of the two related viruses.

## Introduction

Viral neuropathology is often associated with viral access to the central nervous system (CNS) via interaction with brain barriers such as the blood–brain barrier (BBB) and the blood-cerebrospinal fluid barrier (BCSFB) [1,2]. Interactions with brain barriers are crucial for viral access but also induced-pathology as in some cases barrier modulation and dysfunction have deleterious effects on brain homeostasis [3,4]. The BBB is located between neuronal capillaries and the CNS, and is composed of specialized endothelial cells that interact tightly with each other through high expression of tight junction (TJ) proteins, but also of pericytes, astrocytes, microglia and neurons, altogether called the neuro-vascular unit [5]. Numerous arboviruses (arthropod-borne viruses) can be associated with neuropathology in humans, triggering severe and long-term forms or in some cases death [6]. Among them, some members of the Flavivirus family lead to neurological disorders such as Japanese encephalitis virus (JEV), Zika virus (ZIKV), in some cases dengue virus (DENV), West Nile virus (WNV) and Usutu virus (USUV) [7–10]. Studies on the interaction between human brain endothelial cells and *Flaviviruses* have demonstrated CNS invasion by direct infection of these cells and suggest that the BBB is a key gateway for arbovirus brain infections. For instance, JEV and ZIKV are able to infect cerebral endothelial cells and cross the BBB [11,12].

The Trojan horse mechanism has been described for several pathogens, which can productively infect cells of the immune system and relies on their innate properties to access the CNS [13]. Indeed, immune cells play a central role in CNS surveillance and function. A tight balance between responses to infections and deleterious effects associated with neuroinflammation highlights ambiguous roles of immune cells during virus nervous spread [14]. During CNS inflammation, cytokines and chemokines will promote cellular neuroinvasion through multiple mechanisms, including upregulation of cell adhesion molecules (CAMs) on CNS barriers that will allow cell attachment and transmigration [15]. Hijacking of such mechanisms has been found for various neurotropic pathogens including some arboviruses such as WNV [2,16]. WNV and USUV are closely related and found associated with severe neurological symptoms [10,17]. As many others arboviruses, WNV and USUV have emerged from Africa and expanded to Europe where they became endemic. Indeed, they share a similar enzootic cycle involving birds as reservoirs and mainly *Culex* mosquitoes as vectors [18]. The risk of human epidemic is also increasing and lot of efforts are being made to investigate virus distribution and molecular characteristics [19,20]. Neurological disorders associated to human USUV and WNV infections are caused by efficient viral neurotropism. Indeed, human cases of USUV and WNV infections have been associated to neurological impairments such as meningitis, encephalitis, meningoencephalitis, acute flaccid paralysis or Guillain-Barré syndrome. It can be noted that these manifestations are most of the time associated to USUV Europe 2 strain and WNV lineage 2 [17]. Notably, we have previously shown that the Europe 2 strain of USUV was the most neurovirulent *in vitro* and *in vivo* among 6 USUV lineages [21].

While both viruses have been found to infect brain cells including neurons and glia, the mechanisms behind CNS entry have been mainly described for WNV. Indeed, WNV can infect directly BBB endothelial cells or use infected monocytes to reach the CNS [22–26]. Moreover, the presence of WNV and pathogen-associated molecular patterns (PAMPs) in the blood can also lead to BBB dysregulation with a decrease of TJ protein expression and viral transcytosis across the BBB [27]. Through recognition of these PAMPs by pattern-recognition receptors (PRRs), flaviviruses in general, and WNV in particular can induce an antiviral response leading to the secretion of interferons (IFNs) and inflammatory cytokines (e.g. type-I IFN, TNF-α, IL-1β, IL-6, IL-10) [28]. These mechanisms, besides the control of viral propagation, can participate in systemic inflammation and exacerbate clinical onset. However, little is known regarding the mechanisms behind CNS access and antiviral responses of USUV. Interestingly, studies suggest that monocytes and dendritic cells (DCs) are differentially targeted by USUV and WNV during initial infection at the dermal site [29]. Whether this class of cells or their infected status could play a different role in CNS invasion of both viruses remained to be tested.

Here, we compared neuroinvasion and neuroinflammation capacities of these two flaviviruses by analysing USUV Europe 2 and WNV lineage 2 (associated to neurological disease in humans) infection rates, inflammatory responses and the role of monocytes and DCs in virus-BBB interaction.

## Materials and methods

### Cell culture and antibodies

C6/36 cells (Aedes albopictus cells, ATCC CRL-1660) were maintained at 28°C in Roswell Park Memorial Institute culture medium (RPMI, from Eurobio) supplemented with 10% (during maintenance period) or 2% (to avoid serum protein interference at receptor surface) of heat-inactivated fetal bovine serum (FBS), 100 µg/mL streptomycin and 100 U penicillin. Vero cells (African green monkey kidney cells, ATCC, USA) were maintained at 37°C with 5% CO_2_ in Dulbecco’s modified Eagle’s medium (DMEM, from PanBiotech) supplemented with 10 or 2% of heat-inactivated FBS, 100 µg/mL streptomycin and 100 U penicillin. Human brain microvascular endothelial cells (CECs, catalog #1000, ScienCell) were maintained on fibronectin-coated plates according to manufacturer’s instructions and used between passage 2 and 4. T cells, monocytes and monocyte-derived dendritic cells (MoDCs) were obtained from FICOLL purified peripheral blood mononuclear cells (PBMC) using standard protocols.

For this study, mouse anti-E-selectin (BBA1, R&D systems); mouse anti-ICAM-1 (BBA3, R&D systems); mouse anti-pan-flavivirus (clone 4G2, MAB10216; Millipore); mouse anti-VCAM-1 (BBA5, R&D systems); rabbit anti-ZO-1 (617300; Invitrogen); rabbit anti-β-catenin (clone E247, Abcam); human HLA-DR-APC (clone REA805, Miltenyi), mouse anti-CD80-PE (clone 2D10, Miltenyi), human anti-CD83-FITC (clone REA714, Miltenyi) and mouse anti-DC-SIGN-PE (clone DCN47.5, Miltenyi), Hoescht (from Sigma) and ActinGreen (R37110, Thermo Fisher Scientific) were used.

### Viral strains

Usutu Europe 2 strain (TE20421/Italy/2017) was provided from Instituto Zooprofilattico Sperimentale, Emilia Romagna (Italy) and West-Nile lineage 2 (WNV-3125/France/2018) was provided by ANSES (National Agency for Food, Environment and Occupational Health Safety, France). These strains were amplified in limit of four times on Vero cells by infecting 70% confluent cells and collecting supernatants between 5 and 7 dpi. Viral titres were determined using Spearman-Kärber method and were expressed as 50% tissue culture infective dose per millilitre (TCID50/mL) [30]. Infections were done on cells at sub confluence by adding viral or mock-infected inoculum in a small volume of medium during 2 h (h) at 37°C under agitation before removing inoculum and adding fresh medium.

### In vitro human-BBB model

Human umbilical cord bloods were collected after infant’s parents signed consent form in compliance with French legislation. From these and following previously published protocol [12]. CD34^+^ blood-derived endothelial cells were cultured on matrigel-coated transwell filters (Costar, 0.4 µm). These cells were placed on top of bovine brain pericytes during 5–7 days allowing differentiation to human brain-like endothelial cells (hBLECs) that achieved human BBB characteristics as tight-junction proteins and transporters expression [31,32]. During this period, medium was changed every 2 days and before experiments the endothelial permeability coefficient (Pe) was measured using the Lucifer Yellow (LY) (Life Technologies, 20 µM). Before and after experiments on the human BBB model, Pe was measured after screening of paracellular passage of LY by fluorescence detection on Tecan SPARK 10M machine (432/538 nm of excitation/emission wavelength settings). Indeed, the small hydrophilic LY molecule shows a very limited cerebral penetration in physiological conditions. With Pe < 1 × 10^−3^ cm/min, the endothelium was considered impermeable when it was considered to be disturbed with Pe > 1 × 10^−3^ cm/min [33].

USUV or WNV infection of the model was done at multiplicity of infection (MOI 0.1) or 1 for 2 h in limited volume of media low in sera, then viral or mock-infected inoculum were removed and replaced by fresh culture medium. Supernatants were collected at 4, 7 and 10 days post-infection (dpi); and endothelium permeability was measured at 10 dpi. The Spearman-Kärber method was applied to determine viral replication in 4, 7 and 10 dpi supernatants in TCID50/mL [30].

For biomolecular analysis, hBLEC’s RNA were collected at 4 or 7 dpi, whereas for immunofluorescence assays, hBLEC were fixed at 7 or 10 dpi, and for inflammatory analysis, 100 µL of supernatants at 7 dpi were heat-inactivated by incubation in water bath for 30 min at 58°C.

### Human primary cell isolation and differentiation

Buffy coats from healthy donors were obtained from the Etablissement Français du Sang (EFS, Montpellier, France). PBMCs were isolated by density centrifugation using Lymphoprep medium (STEMCELL Technologies). CD4^+^ T lymphocytes were isolated using CD4 MicroBeads (Miltenyi Biotec), activated by phytohemaglutinin (PHA) for 24 hand maintained for 7 days with the cytokine IL-2. CD14 + monocytes were isolated from PBMCs using CD14 MicroBeads (Miltenyi Biotec) and used for experiments and/ or subsequently differentiated into monocyte-derived dendritic cells (MoDCs). MoDCs were generated by incubating purified monocytes in Iscove’s Modified Dulbecco’s Medium (IMDM) supplemented with 10% FBS, 1% P/S, 2 mM L-glutamine, 10 mM Hepes, 1% non-essential amino-acids, 1 mM sodium pyruvate and cytokines GM-CSF (Granulocyte-Macrophage Colony Stimulating Factor, 500 IU/ml) and IL-4 (500 IU/ml), both from Miltenyi Biotec (Cytobox Mo-DC). Immature MoDCs were harvested at day 6 and cell differentiation was estimated by measuring the expression of DC-SIGN, HLA-DR (class II) by flow cytometry.

### Flow cytometry

All cells were fixed with 2% PFA for 30 min prior to surface staining with corresponding antibodies (ICAM, VCAM, E-selectin, HLA-DR-APC, CD80-PE, CD83-FITC and DC-SIGN-PE) for 1 h at 4°C diluted in a PBS/1% BSA/0.05% solution. For flow cytometry analysis, acquisitions were done on a Fortessa cytometer (B Becton Dickinson D), data were collected with FACSDiva software (Becton Dickinson) and were processed with FlowJo software (Treestar Inc.).

### RT-qPCR analyses

Cells were lysed using the RLT buffer (Qiagen), RNA were extracted with RNeasy mini-kit (Qiagen) and cDNA were synthesized by reverse transcription (Omniscript reverse transcriptase, Qiagen). Analyses were done on LightCycler 480 real-time PCR instrument (Roche) using GAPDH, HPRT1 or RPL13A to normalize (refer to Supplemental Table 1 for a complete list of primers used).

### Immunofluorescence assays

Depending on the cell type, specific fibronectin-coated coverslips or matrigel-coated transwell filters were used. At determined dpi, cells were rinsed in PBS, fixed in 4% PFA and permeabilized with 0.1% Triton X-100/PBS during 5 min at room temperature. For 30 min to 1 h, a blocking step was done with 2% bovine serum albumin at room temperature, then incubation of primary and secondary antibodies were performed in blocking solution. Cells were finally incubated with Hoechst (Sigma) and assembled in fluorescent mounting medium (Prolongold, Thermo Fisher), then imaged with Zeiss SP8 confocal microscope (40x or 63x 1.4 NA Plan Apochromat oil-immersion objectives). Excitation wavelengths used are 405 nm (Hoecsht), 488, 561 and 300 nm; pinhole is set to all channels to 1 μm.

### Multiplex assays

A ProcartaPlex Mouse Cytokine and Chemokine Convenience Panel 1A 36-plex and a ProcartaPlex Human Inflammation Panel 20-plex (Thermo Fisher Scientific) were used according to manufacturer’s procedure to quantify mouse and human inflammatory factors in mouse sera and human BBB supernatants. Records were done with Luminex apparatus (MAGPIX; Thermo Fisher Scientific) and analysed on Prism software (Graphpad Prism 8).

### Leukocyte binding assay

Immune cells were labelled with carboxyfluorescein succinimidyl ester (CFSE) according to manufacturer’s instructions (C34554, Invitrogen) then added on hBLECs for 30 min. After binding, cells were rinsed in PBS, transwell filters fixed in 4% PFA and immunofluorescence assays were performed. Then, the immune cell count of ten different observatory areas, for each experiment, was determined.

### Mouse experiments

Adult *Ifnar*^−*/*−^ mice provided by Mireia Pelegrin from Institute for Regenerative Medicine and Biotherapy (IRMB) were bred and maintained in biosafety level 3 (BSL-3) animal facility according to the French Ministry of Agriculture and European institutional guidelines (Appendix A STE no. 123). 10^3^ TCID50/ mice of USUV, WNV or equivalent amount of PBS were subdermally injected. When mice presented health deterioration or at defined days post infection, they were euthanized by cervical dislocation, sera were collected and frozen at −80°C, and organs (as brains) were collected, fixed in 4% PFA and cut with a microtome (3 µm sections) at RHEM facilities (Montpellier).

### Statistical analysis

A minimum of three independent experiments were analysed with *t* tests (Student *t* test or unpaired Mann–Whitney) on Prism software (Graphpad Prism 8).

### Ethics approval and consent to participate

Mice were bred and maintained according to the European institutional guidelines (Appendix A STE n°123) and the French Ministry of Agriculture. Experiments were approved by the French ethics committee (approval N° 6773-201609161356607). The *in vitro* human BBB model requires the use of human cells obtained from donors. All parents signed an informed consent form, according to the French legislation (CODECOH Number DC2011-1321).

## Results

### Differential sensitivity of the human BBB to USUV and WNV infection

**Figure 1.**
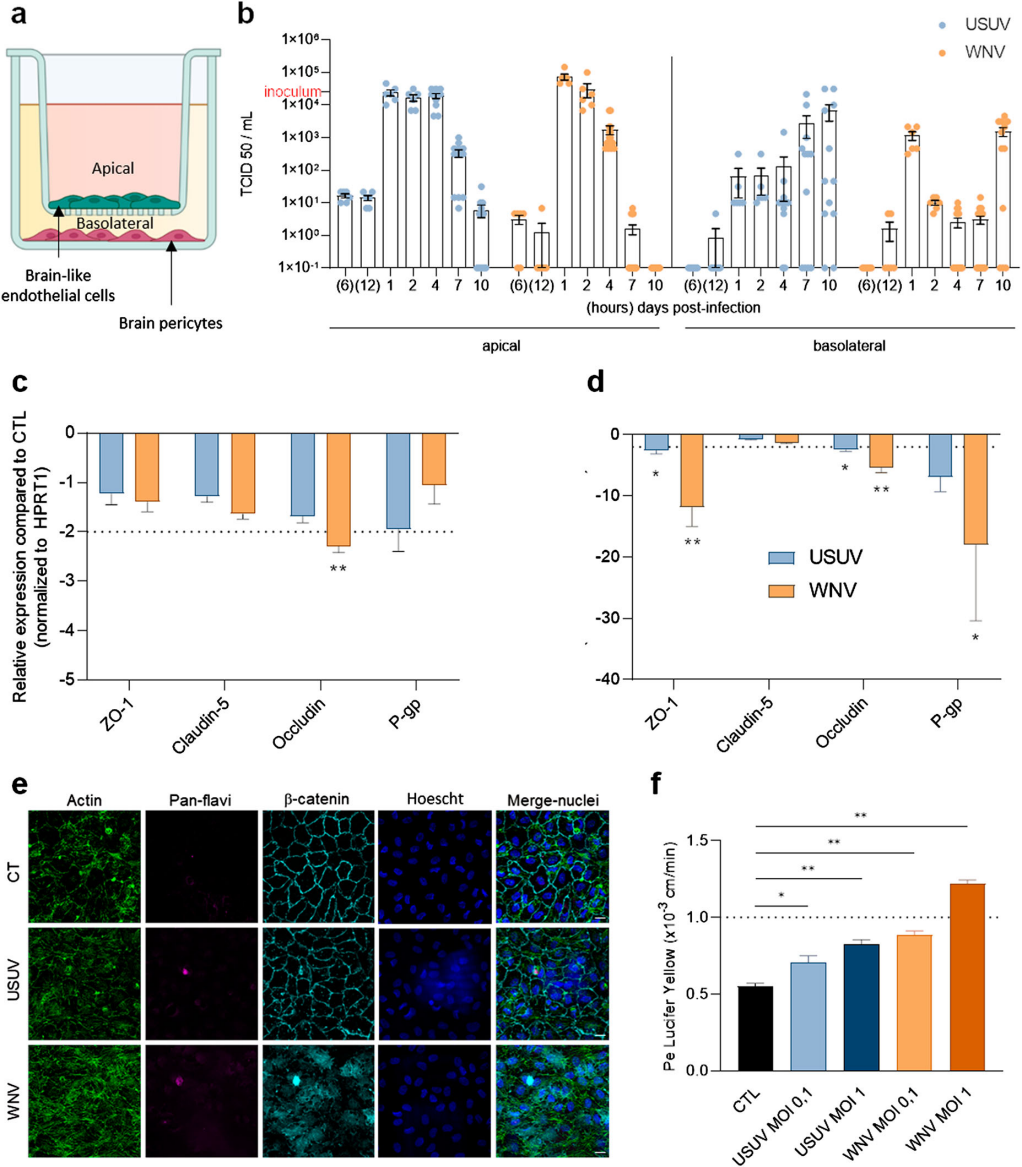
USUV and WNV differentially impact the human BBB integrity *in vitro*. (a) The human *in vitro* BBB model used in this study is composed of brain pericytes (basolateral compartment) allowing the differentiation of CD34^+^-derived endothelial cells towards hBLEC on transwell filters (apical compartment). (b) After 2 h infection of hBLECs (MOI of 0.1, inoculum represented on Y axis), cell were rinsed and refilled with fresh medium, supernatants from apical and basolateral compartments were collected at 6 and 12 h post-infection, then at 1, 2, 4, 7 and 10 dpi; USUV (blue dots) and WNV (orange dots) replication was determined using the TCID50 method. Results are expressed as mean ± SEM (n = 12, from six independent experiments). (c) At 7 dpi, mRNA of mock, USUV or WNV-infected (MOI of 0.1) hBLECs were collected. The expression of tight junction factors and transporters were normalized to housekeeping gene *HPRT1* and compared to mock-infected hBLECs (CTL) in the context of USUV (blue bar chart) and WNV (orange bar chart) infection. Results are expressed as mean ± SEM (*n* = 6; * *p* < 0.05, ** *p* < 0.01, from three independent experiments). (d) Infected- or mock-infected hBLECs (MOI of 0.1) were fixed after 10 dpi and indirect immunofluorescence assays performed to show hBLECs structure with actin (green), viral replication with pan-flavivirus (magenta), junctions with β-catenin labelling (cyan) and nuclei with Hoescht (blue). Scale bar 10 µm. (e) The permeability coefficient (Pe) was measured using the Lucifer Yellow transport assay at 10 dpi with USUV at MOI 0.1 (blue), USUV MOI 1 (dark blue), WNV MOI 0.1 (orange), WNV MOI 1 (dark orange) and mock-infected CTL (black). Bars represent mean ± SEM (*n* = 6; * *p* < 0.05, ** *p* < 0.01, from three independent experiments).

To determine/evaluate the susceptibility of the human BBB to USUV and WNV infection, we took advantage of an *in vitro* BBB model used to study arbovirus-BBB interaction [12]. For this purpose, human CD34^+^ cord blood-derived haematopoietic stem cells were differentiated into hBLECs and seeded on culture inserts with brain pericytes for 5–7 days to acquire BBB characteristics ([34], Figure 1(a)). To monitor and compare USUV and WNV neuroinvasion and neurovirulence, we used the USUV Europe 2 strain and the WNV lineage 2, which are reported with important neurological effects in patients [35,36].

To evaluate viral replication, we infected hBLEC at multiplicity of infection (MOI) 0.1 during 2 h, removed the inoculum, rinsed and replaced fresh medium, and collected supernatants at different time points between 6 h post-infection and 10 dpi. By using the TCID50/mL method, we observed a similar viral replication rate between 1 and 2 dpi, then a higher replication/release of USUV in apical compartment (corresponding to the BBB blood side), as compared with WNV at 4, 7 and 10 dpi (Figure 1(b)). At 1 dpi, WNV seems to be more importantly present in basal compartment (corresponding to the BBB CNS side) compared to USUV, but USUV remains at more higher concentration for 2 dpi until 10 dpi (Figure 1(b)). To confirm these data, we quantified viral RNA in hBLECs and in supernatants and found similar profile in cells for both virus, associated to high viral RNA in apical supernatant at 4 dpi then more important concentration in basal compartment at 10 dpi (Supplemental Figure 1a). We used also another source of primary human cerebral endothelial cells (CEC), which were infected by either USUV or WNV at a MOI of 0.1. At 4 dpi, USUV titre was 1 × 10^3^ TCID50/mL, whereas we observed a titre of 1 × 10^2^ TCID50/mL for WNV, with a decrease in viral titres at 7 dpi (Supplemental Figure 1b), confirming the permissiveness of this cell type to both viruses.

Using multiple approaches, we then assayed the effects of USUV and WNV infections on BBB integrity. We first performed similar experiments at MOI 0.1 and harvested mRNA at 4 and 7 dpi to monitor changes in gene expression of key factors involved in cell–cell interaction regulation (*i.e* TJ and adherens junction factors) and BBB homeostasis (e.g. efflux proteins). We could show that the mRNA expression of these proteins was affected by the two viruses mostly at 7 dpi, with greater proportion in the case of WNV infection (Figure 1(c and d)). The mRNA expression of ZO-1 and Occludin, two TJ proteins that are key elements of BBB integrity [37,38], were the most affected both in USUV and WNV-infected hBLECs compared to mock-infected controls (Figure 1(d)). Similarly, the mRNA expression of P-glycoprotein (an efflux transporter, key for BBB function, P-gp) was found strongly reduced in infected-BBB, primarily for WNV, compared to controls (Figure 1(d)). Moreover, by imaging hBLEC at 10 dpi using confocal microscopy, we were also able to show β-catenin and actin disorganization but no nucleus integrity change following WNV infection whereas the BBB structure remained similar to control with USUV infection (Figure 1(e)). To further determine whether BBB integrity could be impacted by USUV and WNV infection, we evaluated Lucifer Yellow (LY) transport across the BBB model, which can indicate changes in BBB permeability (Figure 1(d)). At 10 dpi, the permeability coefficient (Pe) in mock-infected controls was around 0.5–0.6, which corresponds to a “tight” BBB. Significant Pe increases were detected after USUV infection at MOI 0.1 and 1 translating subtle modulation, but still consistent with an impermeable barrier. Notably, higher Pe was observed in WNV-infected BBB at MOI 1 indicative of a permeable BBB (Figure 1(d)).

Altogether, these results suggest a differential BBB sensitivity to USUV and WNV infections. USUV infection of hBLECs leads to important viral replication and moderate perturbation of endothelium permeability, whereas WNV infection shows more important perturbation despite a lower replication rate.

### USUV and WNV trigger strong inflammatory responses in infected-human BBB

**Figure 2.**
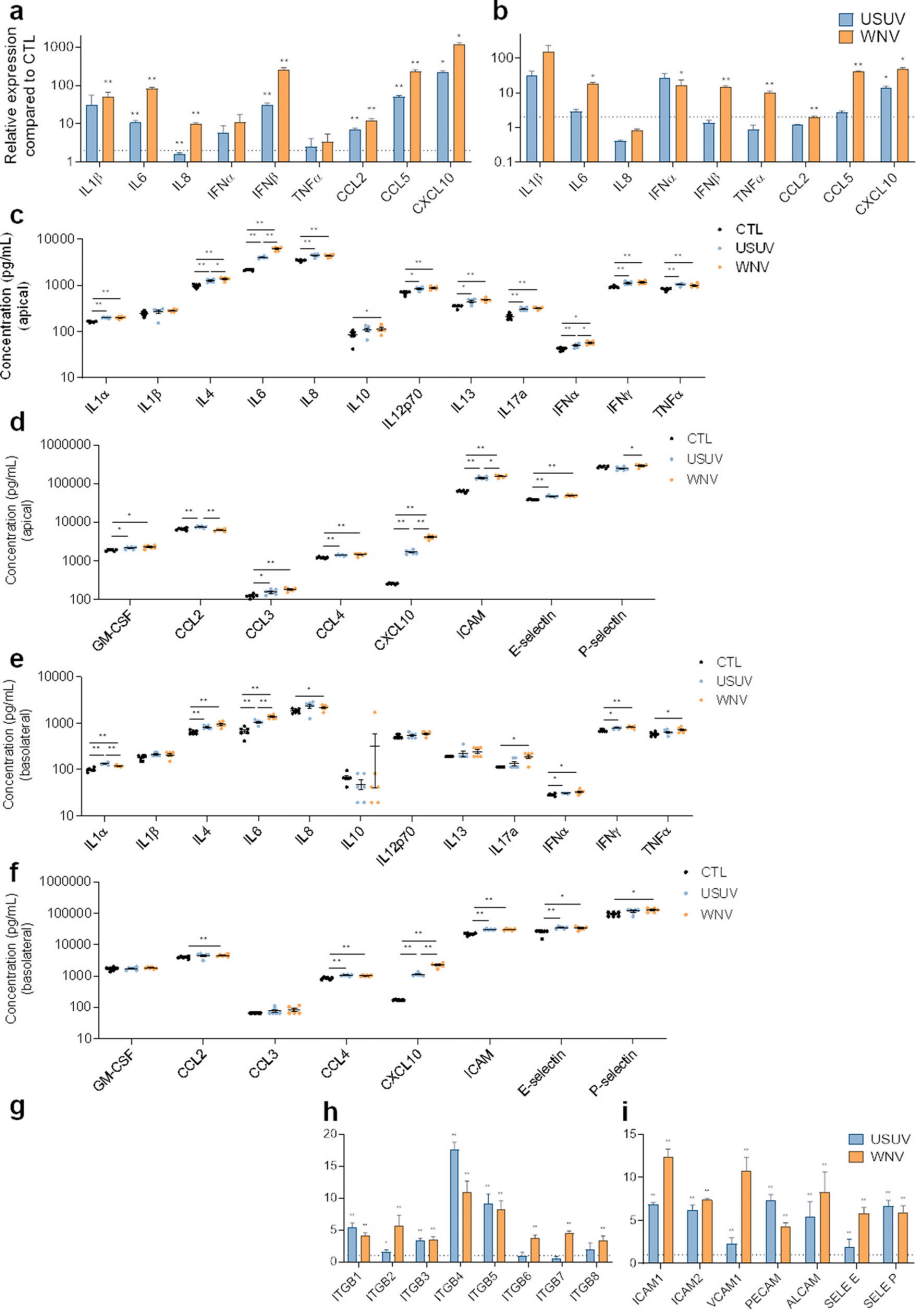
Characterization of innate immune response and CAMs regulation in USUV- and WNV-infected hBLECs. a, b After infection of hBLECs (MOI of 0.1), mRNAs were collected at 4 (a) or at 7 dpi (b). Gene expression was normalized to *HPRT1* and compared to mock-infected hBLECs (CTL) in the context of USUV (blue bar chart) and WNV (orange bar chart) infection. Transcripts of a panel of genes were quantified by RT-qPCR. Data of each indicated transcript are represented as mean ± SEM, relative to mock-infected cells (*n* = 6; * *p* < 0.05, ** *p* < 0.01, from three independent experiments). c, d, e and f Supernatants from apical (c and d) or basolateral (e and f) compartments were collected at 7 dpi and secreted molecules were measured using a multiplexed ELISA assay. Concentrations of cytokines and interferons (c and e) or of chemokines, attractant growth factors and CAMs (d and f) for mock-CTL (black dots), USUV (blue dots) and WNV (orange dots)-infected conditions are represented, bars show mean ± SEM (*n* = 6; * *p* < 0.05, ** *p* < 0.01, from three independent experiments). g, h and i After infection of hBLECs (MOI of 0.1), mRNAs were collected at 7 dpi. Gene expression of α-integrins (g), β-integrins (h), CAMs and selectins (i) were normalized to *RPL13A* and compared to mock-infected hBLECs in the context of USUV (blue bar chart) and WNV (orange bar chart) infections. Results are expressed as mean ± SEM (*n* = 6; * *p* < 0.05, ** *p* < 0.01, from three independent experiments).

USUV and WNV infections are generally associated with antiviral responses and potent inflammation [29]. To monitor the potential differences and/or similarity in BBB infection, we analysed mRNA and protein expression of key cytokines and chemokines in infected hBBB (MOI 0.1) from hBLECs and supernatants at different times post-infection. At 4 dpi, we noticed an upregulation of mRNA expression of several pro-inflammatory and anti-viral molecules (Figure 2(a)). Indeed, hBBB-WNV infection induced a significantly increased expression of interleukins (*IL1β, IL6* and *IL8*), interferon-β (*IFNβ*), tumour necrosis factor-α *(TNF-α)* and chemokines (*CCL2* and *CCL5*) (Figure 2(a)). The establishment of this inflammatory and anti-viral state in hBLECs persisted until 7 dpi with significant up-regulation of *IL6*, *IFNα* and *β*, *CCL2* and *CCL5* (Figure 2(b)). Generally, USUV infection induced a similar, albeit less important, mRNA up-regulation than WNV (Figure 2(a and b)). Both viruses also induced inflammatory and anti-viral responses as shown at 4 dpi by the significant increased expression of *IL1β, IL6, IL8, IFNβ, CCL2* and *CCL5* in the other endothelial cell model (CEC) (Supplemental Figure 1c).

We then quantified secreted proteins in hBBB supernatants at 7 dpi by a multiplexed approach. In the apical compartment, we measured significant elevated concentration of molecules related to anti-viral response and inflammatory state (Figure 2(c and d)). USUV and WNV infections lead to up-secretion of interleukins that can act on the BBB integrity as IL1α, IL6, TNF-α; and up-secretion of chemotaxis factors as GM-CSF, CCL3, CCL4 and CXCL10. In the basolateral compartment, we could detect increased protein expression of IL1α, IL8, IL17a (Figure 2(e)), and CXCL10 or CCL4 (Figure 2(f)). However, we did observe some differences and in particular the fact that the response to WNV seems to be stronger overall. The increase of IL4, IL6 and IFNα was greater for WNV, translating a better capacity to induce pro-inflammatory response. Similarly, CXCL10 was more upregulated following WNV infection. In contrast, USUV infection induced significantly greater upregulation of CCL2 and IL1α.

Another aspect of the immune response to viral infections is the capacity of infected cells to recruit and interact with immune cells to clear viruses. We found upregulation of soluble ICAM-1 (which reflects total cellular ICAM-1) (WNV›USUV) and E-selectin in the apical and basolateral compartments (Figure 2(d and f)). We also monitored CAMs mRNA expression at 7 dpi (Figure 2(g–i)). Notably, USUV and WNV induce significantly upregulation of α-integrins (Figure 2(g)), β-integrins (Figure 2(h)), and CAMs (Figure 2(i)).

Altogether, these data showed an important activation of inflammatory responses by both viruses in the hBBB, with the production of cytokines known to modulate BBB integrity, as well as chemokines and CAMs notably involved in immune cell recruitment that are major actors in neuroinflammation. However, we demonstrated some differences, as a higher inflammation following WNV infection, more induction of CXCL10 and ICAM-1 by WNV, in contrast to the more significant induction of CCL2 by USUV.

### Comparison of systemic and brain inflammation following USUV and WNV infections in vivo

**Figure 3.**
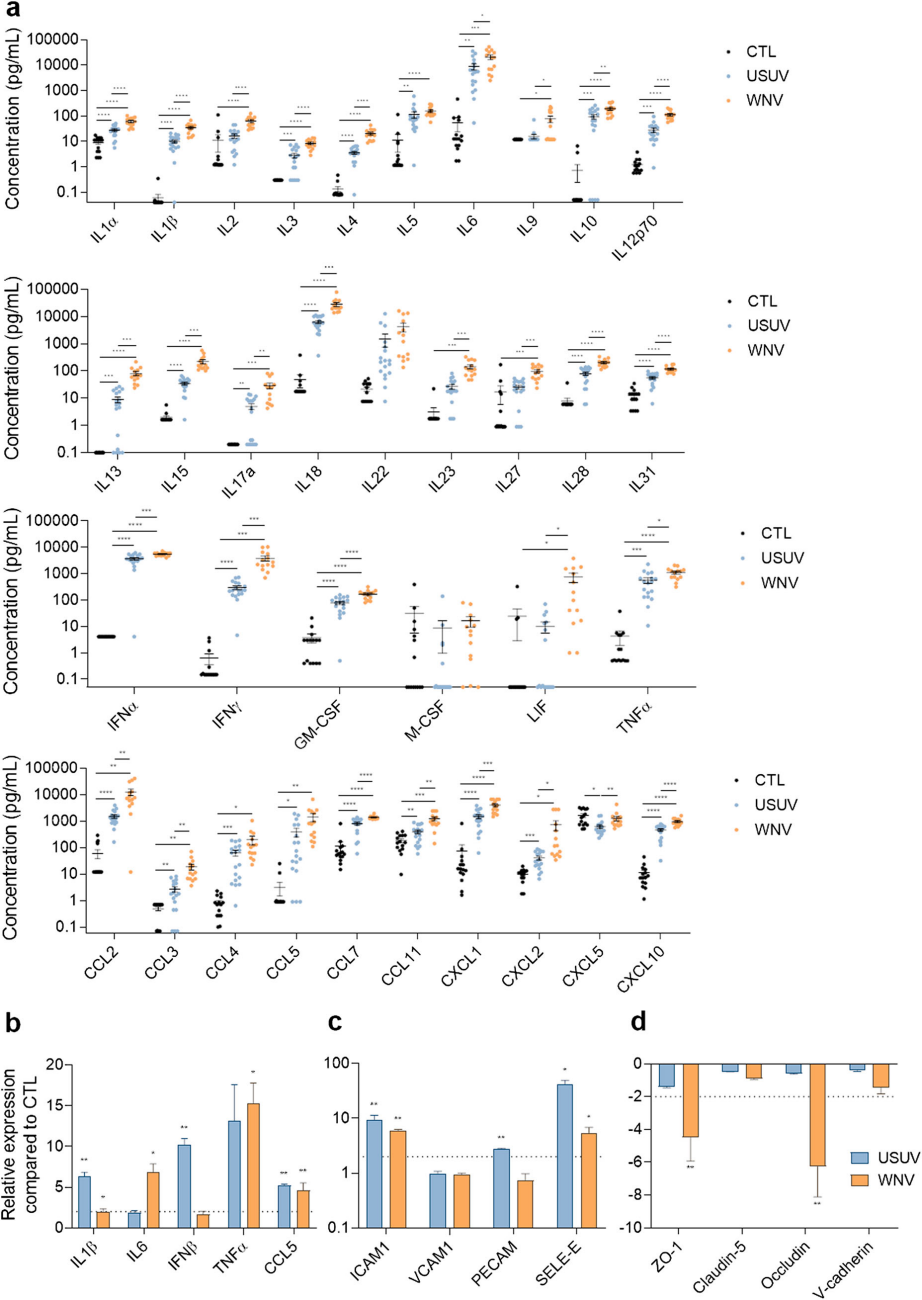
USUV and WNV infection induce different systemic and neuro-inflammation *in vivo* a Mice sera were collected at 2 or 3 dpi and inflammatory molecules were measured using a multiplexed ELISA assay. Concentrations of cytokines, IFNs, attractant growth factors and chemokines from mock-infected (CTL, black dots, *n* = 15), USUV (blue dots, *n* = 18) and WNV (orange dots, *n* = 14) are represented, bars show mean ± SEM (* *p* < 0.05, ** *p* < 0.01, *** *p* < 0.001, **** *p* < 0.0001). b, c, and d After ip infection of *Ifnar^-/-^* mice, brain mRNAs were extracted and purified. Gene expression of anti-viral and pro-inflammatory (b), cell adhesion (c) or tight junction (d) molecules were normalized to *GAPDH* and compared to control mice brain (CTL) in the context of USUV (blue bar chart) and WNV (orange bar chart) infection. Results are expressed as mean ± SEM (n = 6; * *p* < 0.05, ** *p* < 0.01).

Because we detected differential responses and effects of BBB infection by USUV and WNV, we decided to monitor the systemic and CNS inflammation, as well as the potential effects on key BBB proteins *in vivo*. As immunocompetent mice are poorly infected by USUV and manifest low symptoms [39], mice lacking the interferon *α/β* receptor (*Ifnar^-/-^*) were used to investigate USUV and WNV infections. In infected mice, clinical signs and mortality rate were similar between USUV and WNV but WNV-infected mice die more rapidly after infection (around 3 dpi compared to around 5 dpi for USUV) (Supplemental Figure 2b and c). After appearance of symptoms (lethargy and inactivity, limb weakness, ocular defects), mice were euthanized (3–4 dpi), and infection rate was measured in spleen by RT-qPCR (Supplemental Figure 2a). First, systemic inflammation following the infection by these viruses was evaluated with a multiplexed ELISA method. We measured important and significant increased concentrations of several inflammatory factors associated to anti-viral responses (Figure 3(a)). Among them, we found increased concentration of IL1β, IL6, IL10, IL18 or type I and II IFNs (α and γ), TNF-α, chemokines such as CCL2 and CXCL10 and GM-CSF (Figure 3(a)). Generally, WNV infection led to greater inflammatory response than USUV.

Viral genomic RNA was found in the brain of USUV and WNV-infected mice by RT-qPCR analysis (Supplemental Figure 2a). We then measured in the brain the expression of some key inflammatory factors and observed significant upregulation of *IL1β*, *IFNβ* and *CCL5* after USUV infection, whereas *IL6*, *TNF-α* and *CCL5* were significantly upregulated after WNV infection (Figure 3(b)). We also detected increased expression of *ICAM1* and *SELE-E* compared to control animals (Figure 3(c)). Finally, we monitored the expression of key BBB homeostasis factors and detected in WNV-infected mouse brains significant mRNA downregulation of *ZO-1* and *occludin*, whereas in USUV-infected animals this effect was not seen, suggesting that viral effect on BBB integrity could differ between the two viruses (Figure 3(d)).

These *in vivo* results correlated with the previous *in vitro* data showing a more pronounced effect of WNV in systemic inflammation and the production of chemoattractants following both infections, which could potentially lead to immune cell recruitment and CNS infiltration.

### The inflammatory environment of infected-BBB modulates phenotype of monocytes and DCs

**Figure 4.**
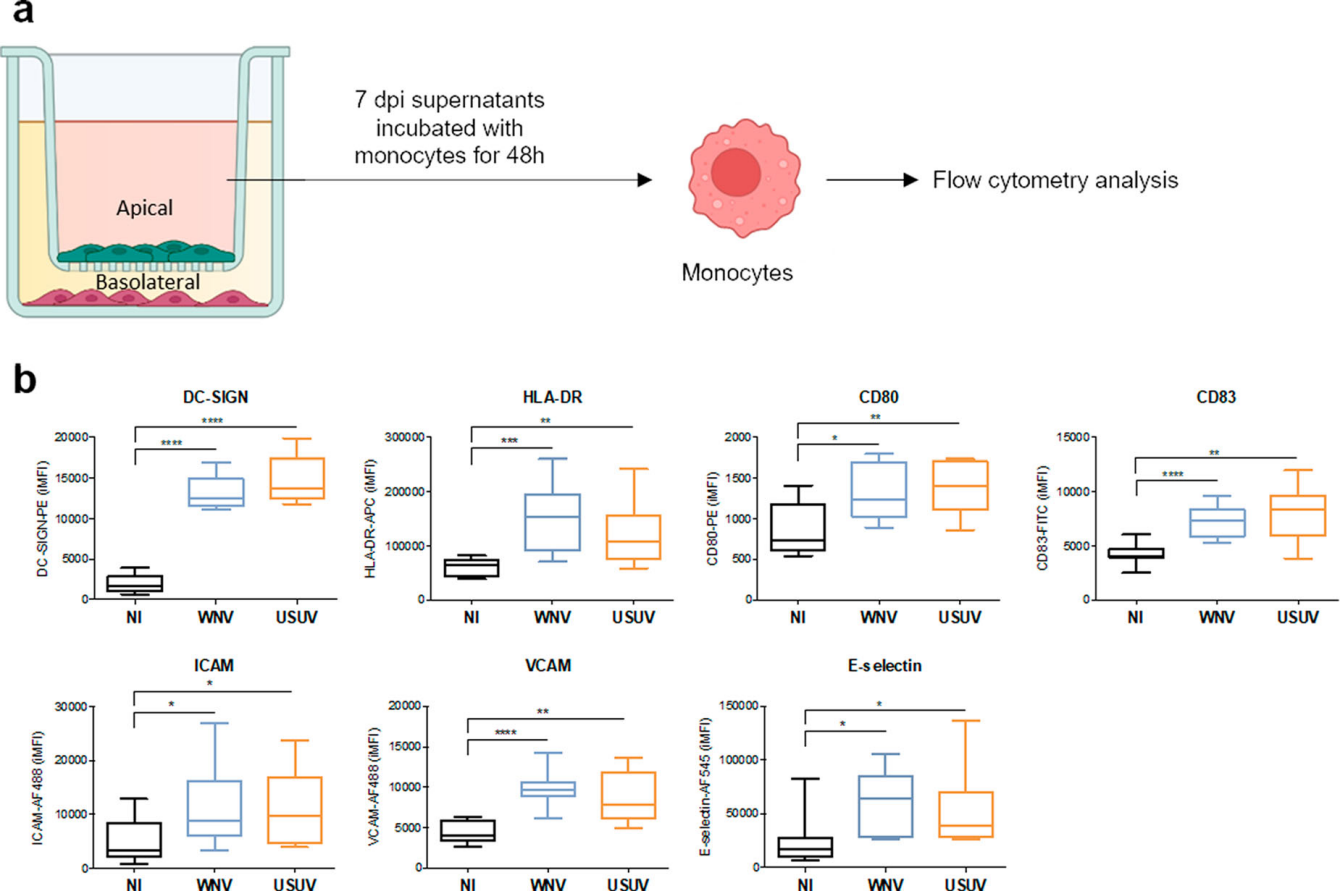
The inflammatory environment of infected-BBB modulates molecular and functional human primary immune cell phenotype a Apical supernatants of mock-, USUV- or WNV-infected hBLECs were collected 7 dpi, and, after viral inactivation by heating at 56°C, incubated for 48 h with monocytes . Cells were analysed by flow cytometry for characteristic membrane factors. b Integrated mean fluorescence intensity (iMFI) of DC-SIGN, HLA-DR, CD80, CD83 ICAM, VCAM and E-selectin, on monocyte from three independent experiments are represented as box and whiskers with median ± min to max. Statistical significance (*p* value) was determined by Student’s *t* test. (*p < 0.05; **p < 0.01; ***p < 0.001, ****p < 0.0001).

Because we observed a strong inflammatory environment coupled to an increase of molecules modulating circulating immune cell recruitment to the BBB, we decided to analyse the potential effect of this environment on immune cells. We focused on monocytes (Mo) since they are primary viral targets in systemic circulation and major actors of the anti-viral response. We incubated heat-inactivated supernatants from the apical compartment (corresponding to the blood side) of USUV- and WNV-infected hBBB at 7 dpi with Mo. After 48 h, we fixed and incubated cells with specific antibodies against key markers of immune cell activation and differentiation (Figure 4(a)). Mo incubated with USUV- or WNV-infected supernatants showed greater expression of ICAM-1, VCAM-1 and E-selectin. They also showed higher expression of maturation and activation factors, including HLA-DR, CD80, CD83 and DC-SIGN (dendritic cell-specific intercellular adhesion molecule-3-grabbing non-integrin), suggesting activation and differentiation into DCs (Figure 4(b)).

These data indicate potential phenotype change of monocytes as well as their possible recruitment at the BBB after USUV or WNV infection.

### USUV and WNV infected-BBB promotes binding of leukocytes

**Figure 5.**
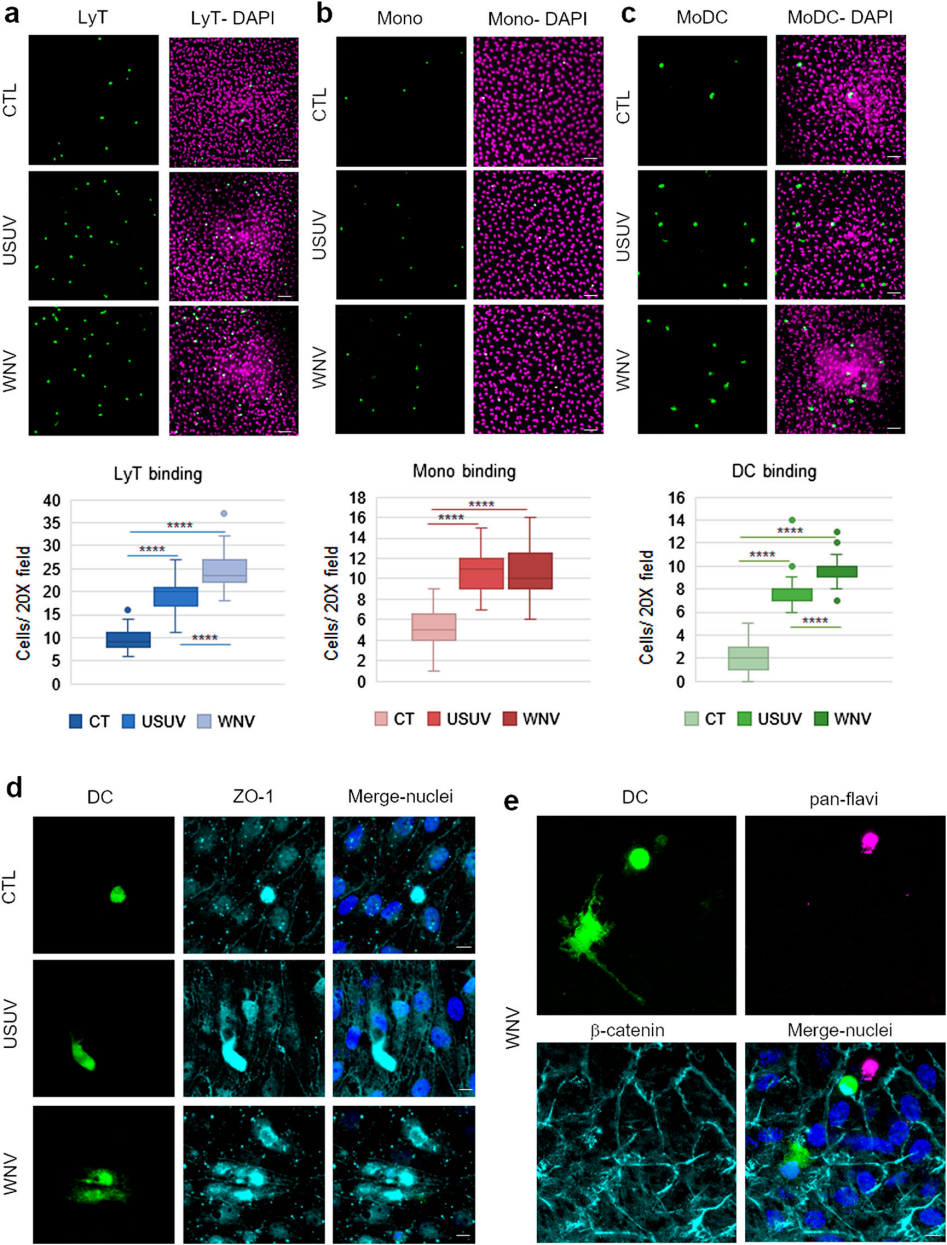
The infected BBB can recruit T cells, monocytes and MoDCs. a, b and c After 7 dpi of hBLECs, CFSE labelled-T cells (a), monocytes (b) or MoDCs (c) were incubated on the apical side of infected-hBLECs during 30 min (for each condition, six independent experiments were analysed from two independent hBLEC infection experiments), cells were rinsed and fixed to analyse number of immune cells bound. Indirect immunofluorescence images show immune cells (LyT, monocytes and MoDCs, in green) and hBLECs nucleus (magenta). Scale bar 50 µm. Box plots represent quantitative analyses of immune cell per field (20x) in CTL, USUV or WNV-infected hBBB (**** *p* < 0.0001). d CFSE-labelled MoDCs show cell spreading on infected-hBLECs (ZO-1, cyan; nucleus, blue). Scale bar 10 µm. e On WNV-infected BBB (pan-flavivirus in magenta), MoDCs (green) bind to hBLECs (β-catenin, cyan; nucleus, blue) and are displaying change in morphology consistent with cell spreading. Scale bar 10 µm.

Since USUV- and WNV-infected-BBB displayed upregulation of inflammatory, chemotaxis and cell interaction factors, we then assessed leukocyte binding on infected-hBBB. At 7 dpi, we incubated CFSE-labelled PBMC-purified human lymphocytes T CD4^+^ (LyT), CFSE-labelled PBMC-purified monocytes or or CFSE-labelled PBMC-derived MoDCs for 30 min on the hBBB. We could observe a significant increase of LyT recruitment on USUV- and WNV-infected hBBB (Figure 5(a)). Infection also led to significant increase of binding of monocytes (Figure 5(b)), and MoDCs (Figure 5(c)). Moreover, recruited MoDC displayed differential morphology after binding to USUV and WNV-infected hBLECs (Figure 5(d and e)). These observations could be consistent with the beginning of transmigration across the infected-BBB.

This set of data confirm that USUV- and WNV-infected hBBB could potentially attract and recruit circulating immune cells during the course of infection. Both viruses led to an increased binding of LyT, monocytes and MoDCs at the BBB, while WNV infection led to significantly greater recruitment of LyT and MoDCs than USUV.

### Monocyte and DC infection potentiates hBBB binding

**Figure 6.**
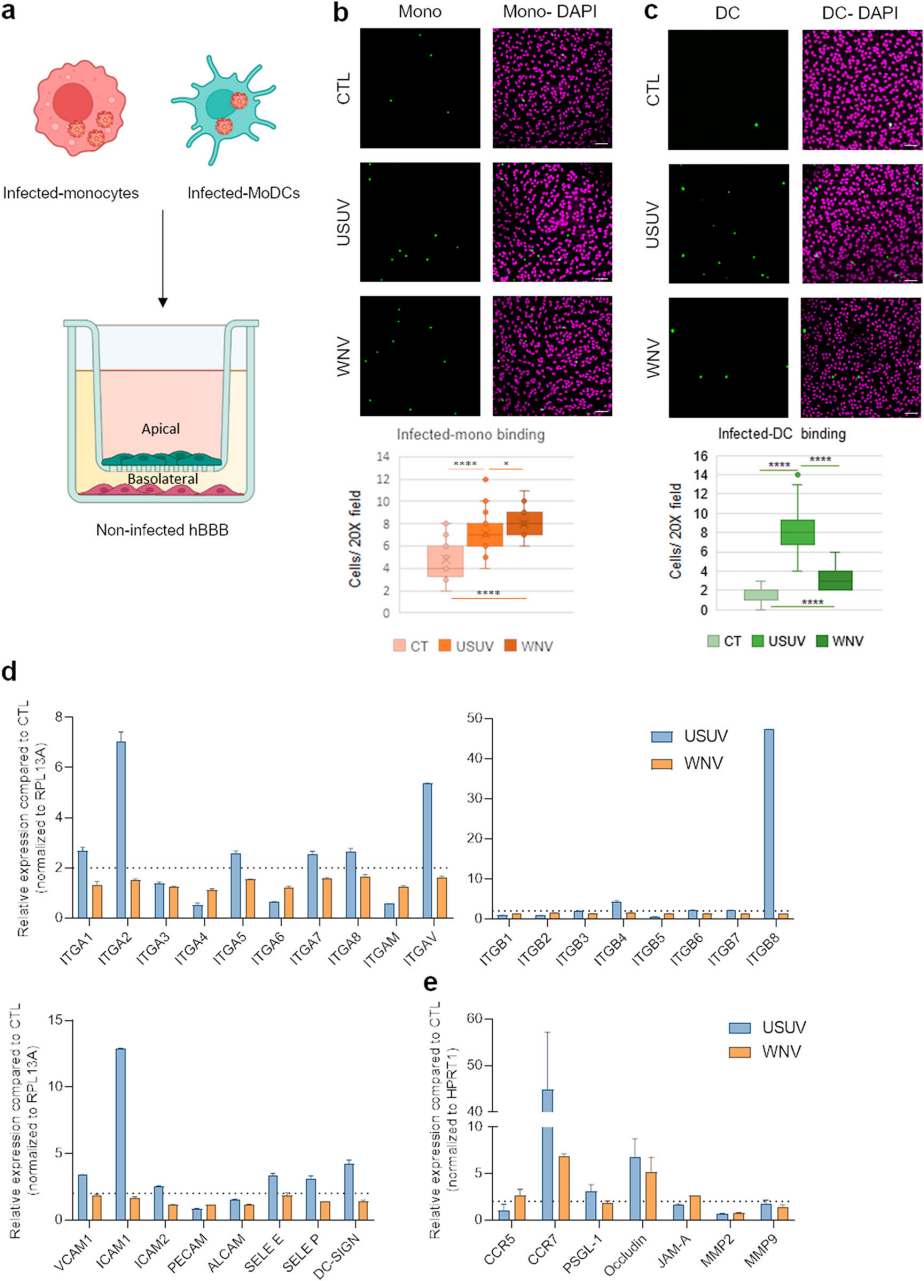
Infected-monocytes and DCs interact with the human BBB. a Monocytes and MoDCs were infected with USUV or WNV at MOI 0.1 for 48 h and CFSE-labelled before being added on hBLECs for 30 min (for each condition, three independent experiments were analysed). hBLECs were rinsed, fixed, and analysed by microscopy. b and c CFSE labelled- and infected-monocytes (b) or MoDCs (c) were incubated on hBLECs. Indirect immunofluorescence images show immune cells (in green) and hBLECs nucleus (magenta). Scale bar 50 µm. Box plots represent quantitative analyses of CTL, USUV or WNV-infected monocyte numbers per field (20x) (*p < 0.05, **** *p* < 0.0001). d After 48 h of infection, MoDC mRNA were extracted. Gene expression was normalized to *RPL13A* and compared to mock-infected MoDCs (CTL) in the context of USUV (blue bar chart) and WNV (orange bar chart) infection. Results are expressed as mean ± SEM (*n* = 6, from three independent experiments). e After 48 h of infection, MoDC mRNA was extracted. Gene expression was normalized to *GAPDH* and compared to mock-infected MoDCs (CTL) in the context of USUV (blue bar chart) and WNV (orange bar chart) infection. Results are expressed as mean ± SEM (n = 6, from three independent experiments).

CNS transmigration of infected-immune cells, in a process called “Trojan horse” mechanism, has been described for several viruses including WNV, which has been shown to access the brain through infected monocytes [24]. To monitor whether USUV could use similar mechanisms, we analysed hBLEC binding of USUV and WNV-infected purified human monocytes. Cells were infected for 48 h (Supplemental Figure 2b) and allowed to bind to hBLEC for 30 min (Figure 6(a)). In this condition, we could detect significant increased binding of both USUV- and WNV-infected monocytes to non-infected hBLEC (Figure 6(b)), similarly than what we observed for non-infected monocyte binding to infected-hBLECs.

Human arboviral infections occur initially at the epidermis or dermal site at the mosquito bite site. At this stage, the first infected cells can be the local immune cells such as Langerhans and dendritic cells. Therefore, we first measured the response of MoDC to USUV and WNV infections. Cells were infected with USUV and WNV for 48 h (Supplemental Figure 2a) and mRNA harvested. Cells were incubated for 30 min on hBLEC, rinsed and fixed prior immunofluorescence labelling and imaging. We observed significant higher binding of USUV-infected MoDC compared to WNV-infected MoDC (Figure 6(c)).

RT-qPCR analyses showed that USUV infection leads to the upregulation of mRNA expression of integrins and CAMs in a more pronounced manner than WNV (Figure 6(d)). Interestingly, DCs have been shown to express some migratory factors and TJ proteins that could facilitate passage across endothelium or epithelium [40]. We therefore monitored whether infection could modulate the expression of such factors. We observed that USUV and WNV infection induced over-expression of CCR7 and tight junction protein (Occludin) (Figure 6(e)) that can facilitate recruitment and transmigration across the BBB.

Altogether, this set of data suggests that DCs respond differently during USUV and WNV infections, and interestingly, could represent a CNS entry pathway during USUV infection. USUV infected-MoDCs express integrins, CAMs, selectins, chemokine receptor and TJ proteins, that can facilitate the transmigration across endothelia, in contrast to WNV infected-DCs.

## Discussion

USUV and WNV are closely related flaviviruses and display several similar epidemiological and molecular features. In particular, CNS infection and impairments are found associated with human infections, with few cases reported for USUV Europe 2 strain in Europe and several thousand cases in America and Europe for WNV lineage 2, which can sometimes prove fatal to patients [41,42]. Interestingly, these two lineages have been associated with emergence and epidemic events in Europe recently, triggering public health concerns with the development of numbers of neurological disease following human infection. This highlights the need of more characterization concerning the neurovirulence of these lineages to anticipate potential increase of human infection cases. Both viruses have been shown to target several CNS cell types including neurons, astrocytes and microglia cells. Importantly, viral CNS replication was associated with local inflammatory responses, thought to potentiate and/or exacerbate neurological impairments [43]. Moreover, studies have reported that USUV and WNV showed differences in permissiveness and associated-inflammatory responses during infection of various immune cells. In MoDC and Langerhans cells for instance, USUV replicates more rapidly, [29], and WNV seems to have developed more resistance mechanisms to counteract immune response [44]. Whether neuroinflammation due to direct (viral-induced) or indirect (immune response) responses differ between the two viruses is still poorly characterized.

### Viral and immune cell neuroinvasion and neuroinflammation

Direct infection of brain endothelial cells has been reported for several arboviruses, including ZIKV, WNV and USUV [2,12,21]. The production of known BBB-destabilization factors during brain infection such as TGF-β, TNF-α, IL6 and IL1β have been shown to perturb BBB permeability [45]. Here we show that *in vitro*, infection by USUV Europe 2 and WNV lineage 2, two lineages associated with neurological impairments in humans, led to the increased release in both apical and basolateral compartments of the human BBB of IL6 and TNF-α, whereas the genetic modulation of *IL1β* led also to an upregulation of mRNA expression. *In vivo*, we could also observe increased serum concentration in USUV-infected *Ifnar^-/-^* mice of IL1β, IL6 and TNF-α. Similarly that was reported for WNV [46], albeit in a much less marked manner, one can hypothesize that inflammatory molecules produced locally during USUV BBB infection could lead to subtle and restricted BBB integrity modulation. Notably, we report here that USUV led only to a slight modulation of hBBB permeability in a similar manner that what we showed for ZIKV [12]. In this regard, USUV led also to a modest but significant genetic downregulation of TJ factors ZO-1 and Occludin *in vitro*. Therefore, albeit USUV infection of *in vitro* hBBB or *in vivo* led to upregulation of inflammatory and destabilizing BBB factors, as well as modest downregulation of TJ protein, important differences exist between the two related flavivirus regarding the global effect on BBB integrity.

Moreover, systemic and CNS inflammation result in an extracellular environment favouring immune cell recruitments to brain barriers, including the BSCFB and BBB [15]. Here, we also show that USUV and WNV BBB direct infection led to the release of potent chemoattractants such as CXCL10, CCL5 and CCL2 *in vitro* and *in vivo*. The interaction between immune cells and the apical side of the BBB will trigger downstream signalling that will favour binding, crawling and transmigration. For instance, binding to ICAM-1 will activate upregulation of intracellular calcium and modulation of Rho/ROCK and actin signalling, leading to disruption of inter-cellular junctions [43]. Here, we showed that USUV- and WNV-infected BBB upregulated expression and secretion of ICAM-1, ICAM-2, VCAM-1, PECAM, ALCAM but also selectins *in vitro*. These observations then correlated with the increased expression of selected CAMs on monocytes and DCs incubated with supernatants from USUV and WNV-infected hBBB and greater leukocyte binding that we observed in USUV- and WNV-infected BBB. Whether the differential upregulation of chemokines and CAMs observed in USUV and WNV infections will lead to different type or quantity of immune cell BBB recruitment and CNS infiltration remain to be fully addressed but our *in vitro* results suggest difference correlated with the quantity of chemoattractant and inflammatory factors released (WNV leading to more T lymphocyte, monocyte and DC recruitment) or the direct infection rate of immune cells (higher USUV infection of DCs leading to higher BBB binding). However, even though the general inflammatory response is more potent for WNV than for USUV, some chemokines such as CCL2 are more secreted by the hBBB upon USUV infection. Whether differences in key inflammatory factors will dictate differential neuroinvasion and neuroinfllammation during viral brain infection will need to be demonstrated. Here, we showed differential viral replication rates between USUV and WNV in our *in vitro* and *in vivo* models. Interestingly, the replication rate did not correlate with the importance of associated-inflammatory responses and endothelium integrity impairment. In other words, we show a higher replication for USUV compared to WNV in hBLEC but a higher inflammatory response and integrity modulation for WNV. *In vivo,* we detected more systemic replication of USUV whereas WNV was showed to replicate more efficiently in the brain. Pro- and anti-inflammatory responses to viral infection need to be thinly regulated, as an imbalance in the antiviral and inflammatory responses can lead to direct damages and/or to the development of long-term impairments. In the case of these two *Flaviviruses*, it seems that if USUV replicates more efficiently, the damages caused by WNV are more important and could be due to the large inflammatory response possibly because it induces a stronger inflammatory response.

These observations suggest that the anti-viral responses against WNV and USUV may vary and prevent or exacerbate differently cell-type associated-deleterious effects that do not particularly correlated with the replication rates.

### A focus on monocytes, DC, Trojan horse and brain infections

Numerous studies regarding WNV neuroinfection showed the key role of circulating leukocytes and monocytes, both in favouring CNS access (Trojan horse mechanism, see below) or in protecting against brain infection [47,48]. Most of the studies have been focusing on the role of CD4 and CD8^+^ T cells but emerging data suggest that monocytes have a key role during the course of the pathology [24,49]. Studies reporting the proliferation of monocytes during WNV infection suggested that this occurred prior brain targeting [50]. However, very little is reported regarding the role of DCs during WNV CNS infection or the immune cells involved in USUV-induced cellular infiltration during brain infection. During inflammation, monocytes can be recruited and differentiate in DCs. Studies suggest that BBB interaction, as well as following transmigration may induce monocyte differentiation towards DCs. For instance, in a mouse model of multiple sclerosis, TGF-β and GM-CSF secreted by the BBB have been shown to induce the differentiation of CD14^+^ BBB-bound monocytes towards CD83^+^CD209^+^ DCs [51]. Interestingly, we found both *in vitro* (hBBB) and *in vivo* (*Ifnar*^-/-^ mice) that GM-CSF concentration was increased upon USUV and WNV infections. Moreover, the local inflammatory environment induced by hBBB USUV and WNV infections led to a change of status of PBMC-derived monocytes, which expressed molecules that sign a differentiation towards DCs. Notably, despite the heat-inactivation of infectious viruses in supernatants used in these experiments, one cannot rule out the potential role of viral proteins in direct immune cell status modulation as it is described in particular for flaviviruses and endothelial cells for instance [52]. Regarding their role as Trojan horse, infected-monocytes have been proposed to act as viral CNS carriers for several viruses including HIV [53]. Similarly, here we show that USUV-infected human primary monocytes also preferentially bind to the hBBB *in vitro* and could represent a CNS access platform for this flavivirus.

DCs are natural sentinels of the brain participating in homeostasis surveillance but they can also potentiate neuroinflammation. For example, during CNS infection they potentiate T cell responses and release pro-inflammatory cytokines [14]. In brain disorders, number of DC in parenchyma and activation status have been found to be correlated with severity of the disease [54]. Under local or systemic inflammatory environment, chemotactic factors such as CCL2 [55] have been shown to favour DC recruitment and lead to their interaction with the BBB through expression of CAMs and their transmigration through the activation of specific adhesion and signalling mechanisms [14]. Interestingly, both the inflammatory environment of USUV- and WNV-infected BBB and the direct infection of DCs by both viruses led to the upregulation of CAMs, in particular DC-SIGN. This was particularly the case for USUV infection. Finally, it was suggested that the expression of some TJ proteins by DCs could facilitate their transmigration across the BBB [40]. These molecules may assist in BBB transcytosis by helping to maintain barrier integrity by forming transient TJ-like structures with surrounding endothelial cells during DC extravasation. For instance, DC subpopulations have been shown to express JAM-A and Occludin, both important cell–cell interaction molecules involved in TJ maintenance [56,57]. Notably, USUV- and WNV-infected DCs showed upregulation of JAM-A and Occludin, which could facilitate transmigration in a similar mechanism. Because USUV led to a better infection of DCs, the upregulation of molecules involved in binding and transmigration was much more pronounced, which could suggest that this type of immune cells may have a more pronounced role during USUV neuroinvasion than for WNV. Very little data exist regarding their role as a potential Trojan horse. Evidence has shown that infected-DCs could participate in Nipah and Toscana virus infection of CNS [16,58]. Here, we show that USUV-infected DCs are preferentially binding to the human BBB *in vitro*. This could be partially explained by a better permissivity for USUV infection and subsequent upregulation of key CAMs, favouring BBB binding. DCs may represent a different or more efficient CNS access platform for USUV compared to WNV.

## Conclusion

Understanding the mechanisms behind neuroinvasion and associated neuroinflammation during arbovirus infection is of interest as specific therapeutical approaches are still missing. Identifying key aspects of brain invasion and potential peripheral biomarkers that could predict severe forms is of crucial importance. Moreover, there is a clear need to assess the difference between closely related viruses and different strains or lineages that can prove more or less (neuro)virulent to anticipate potential emergence of variants of concern for public health.

## Supplementary Material

Supplemental Material
