# Supplementary figures and images for: Differential effects of Usutu and West Nile viruses on neuroinflammation, immune cell recruitment and blood–brain barrier integrity
Source: Emerg Microbes Infect. 2023 Jan 2;12(1):2156815. doi: 10.1080/22221751.2022.2156815 (PMC9815434; doi:10.1080/22221751.2022.2156815)

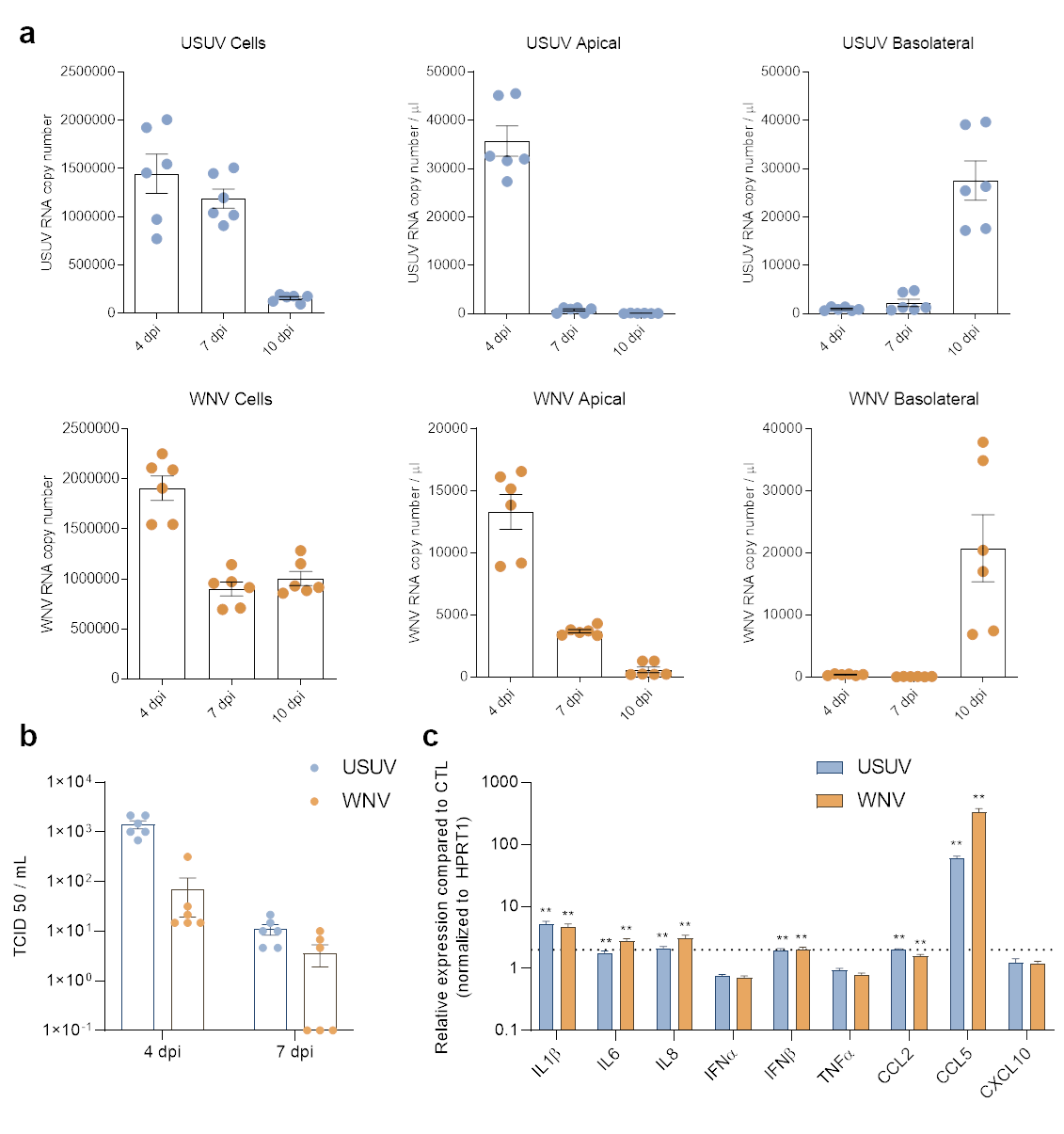

Supplement: Supplemental Material [file TEMI_A_2156815_SM5915.zip › Supplemental Figure 1R.tif]

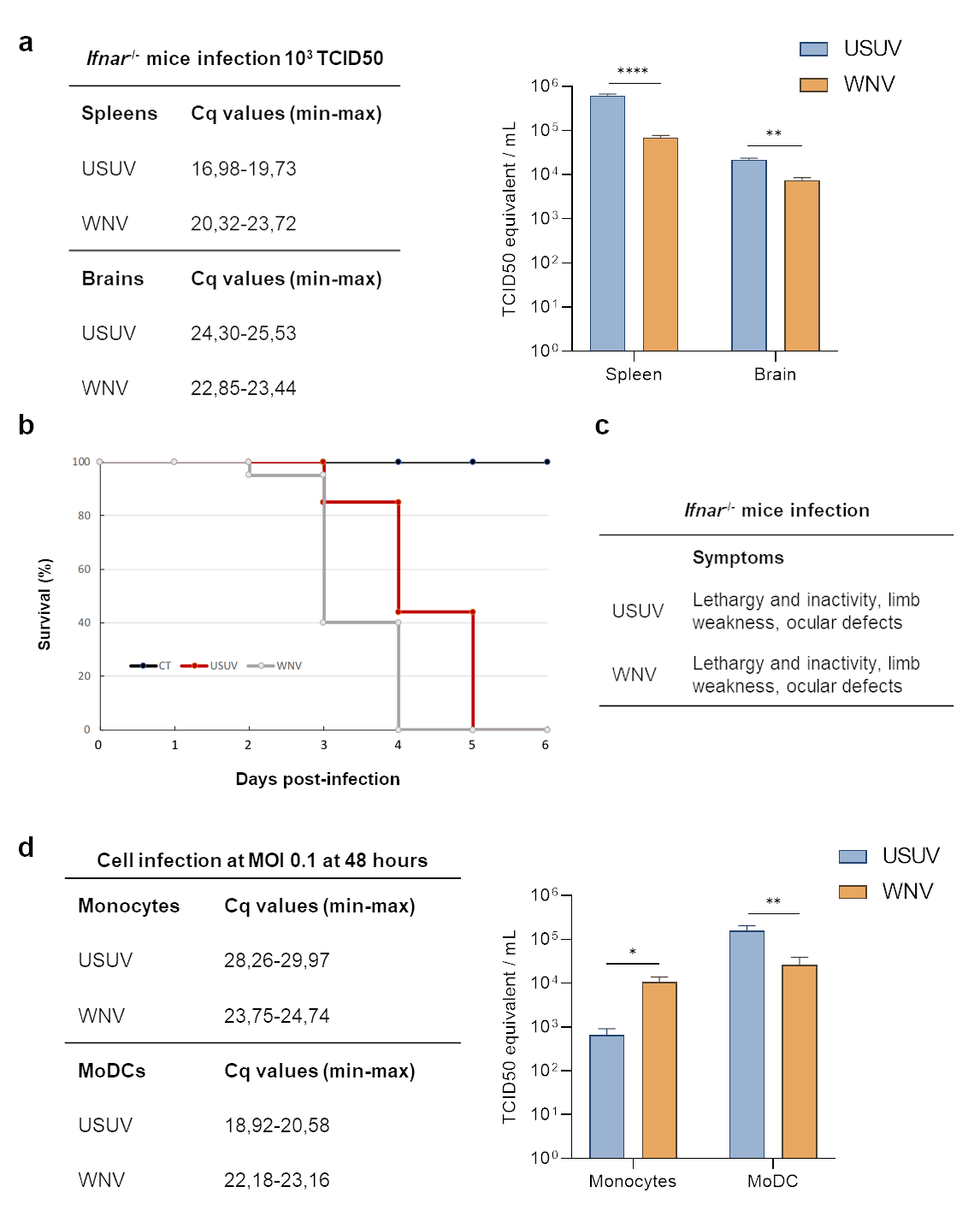

Supplement: Supplemental Material [file TEMI_A_2156815_SM5915.zip › Supplemental Figure 2Rb.tif]
